# Exercise reverses the effects of early life stress on orexin cell reactivity in male but not female rats

**DOI:** 10.3389/fnbeh.2014.00244

**Published:** 2014-07-23

**Authors:** Morgan H. James, Erin J. Campbell, Frederick R. Walker, Doug W. Smith, Heather N. Richardson, Deborah M. Hodgson, Christopher V. Dayas

**Affiliations:** ^1^Neurobiology of Addiction Laboratory, The Centre for Brain and Mental Health Research, School of Biomedical Sciences and Pharmacy, Hunter Medical Research Institute, University of NewcastleNewcastle, NSW, Australia; ^2^Neurobiology of Stress and Addiction Laboratory, Department of Psychology, University of MassachusettsAmherst, MA, USA

**Keywords:** orexin, hypocretin, stress, maternal separation, sex-differences, exercise, plasticity, hypothalamus

## Abstract

Early life stress (ELS) is a known antecedent for the development of mood disorders such as depression. Orexin neurons drive arousal and motivated behaviors in response to stress. We tested the hypothesis that ELS alters orexin system function and leads to an altered stress-induced behavioral phenotype in adulthood. We also investigated if voluntary exercise during adolescent development could reverse the ELS-induced changes. Male and female Wistar rats were subjected to maternal separation stress on postnatal days (PND) 2-14. A subset of animals was given access to running wheels in late adolescence (1hr/day, PND40-70). In adulthood, rats were exposed to restraint stress and then tested on the open field (OF) and elevated plus maze (EPM). Brains were processed for Fos-protein and orexin or tyrosine hydroxylase immunohistochemistry. Restraint stress stimulated Fos-protein expression in perifornical area orexin cells, the paraventricular hypothalamic nucleus, and paraventricular thalamic nuclei, but this neuronal response was dampened in male and female rats exposed to ELS. ELS also reduced exploration in the OF, without affecting EPM behavior. These neural and behavioral changes are consistent with a depressive-like phenotype. Adolescent exercise reversed the orexin and behavioral deficits in ELS males. Exercise was not protective in females, although this may be due to sex differences in running behavior. Our findings highlight the inherent plasticity of the orexin system—a trait that may lead to a state of pathological rewiring but could also be treated using non-pharmacological approaches. We also highlight a need to better understand the sex-specific changes in orexin circuits and stress-related pathology.

## Introduction

Early life stress (ELS) is a major risk factor for the emergence of mood-related disorders such as depression and anxiety in adulthood (Danese et al., 2008). Preclinical studies show that separation of rat pups from their mother during the neonatal period (known as maternal separation) also increases vulnerability to anxiety- and depression-like behavior in adulthood (Winslow and Insel, 1991). The impact of ELS on the brain is dramatic and includes maladaptations to the neuroendocrine hypothalamus (i.e., the paraventricular nucleus; PVN) and associated feedback circuits (Meaney et al., 1996, 2007). Importantly, other hypothalamic systems are known to influence autonomic, neuroendocrine, and behavioral responses to stress, but there have been few studies addressing the impact of ELS on these non-neuroendocrine cell groups. For example, cell groups within the lateral hypothalamus (LH) have the capacity to influence a number of stress-relevant behavioral adaptations, including changes in arousal and reward status (Harris and Aston-Jones, 2006; Furlong et al., 2009). Dysregulation of these LH systems by ELS could significantly increase the risk for development of anxiety and depression in later life.

Of particular interest in this context are the orexin (hypocretin) neurons that are now known to be central to LH-mediated changes in arousal and motivational states (Harris and Aston-Jones, 2006; James et al., 2011, 2012; Johnson et al., 2012). Acute stress robustly increases activation of orexin neurons (Ida et al., 2000; Furlong et al., 2009), whereas chronic stress appears to have an opposite effect (Lutter et al., 2008; Nocjar et al., 2012). The ability of chronic stress to restrict orexin activity is particularly interesting, as evidence has recently emerged linking low orexin system function with depressive symptoms in humans (Brundin et al., 2007, 2009). Surprisingly, the effect of ELS on orexin neuron function in adulthood has not been directly tested. Therefore, the primary aim of this study was to investigate the effects of ELS on orexin system function following psychological stress exposure in adulthood.

Non-pharmacological approaches to produce or augment antidepressant/anxiolytic action have significant clinical relevance and appeal. Both clinical and preclinical studies suggest that physical activity or exercise can produce antidepressant-like effects (Greenwood et al., 2003; Lapmanee et al., 2013). At present however, it is unclear whether the antidepressant or anxiolytic effects of physical activity might be linked to improvement in LH-orexin system function. Thus, a secondary aim of the present study was to investigate the possible preventative effects of physical activity on ELS-induced maladaptive orexin cell responses to stress in adulthood. Finally, because very few studies have examined the sex-specific effects of exercise on stress-related behavior, we carried out our experiments in both male and female rats.

## Methods and materials

### Ethics statement

All procedures performed were approved by the University of Newcastle Animal Care and Ethics Committee, and were carried out in accordance with the New South Wales Animal Research Act.

### Animals

Ten experimentally naïve Wistar dams were obtained from the University of Newcastle Animal house and bred with two experimentally naïve males in the University of Newcastle vivarium. A total of 34 male and 39 female offspring were included in the study. As per previous studies (Caldji et al., 2000; Weaver et al., 2007; Nakamura et al., 2011), litters were not standardized to a fixed number of pups or male/female ratio; rather, these variables were accounted for during data analysis (see Data Analysis section below). On postnatal day 1 (PND1), animals from each litter were randomly allocated to the ELS or control (no ELS) condition. ELS allocated litters underwent maternal separation procedures (detailed below) between PND2-14. On PND21, animals were weaned and separated into same-sex housing, with 2 animals/cage (41.5 × 28 × 22 cm cages; Mascot Wire Works, Sydney). Food (Rat and Mouse Pellets, Glen Forest, Western Australia) and water were available *ad libitum* and rats were maintained on a 12-h light (0600–1800): 12 h dark cycle. Temperature was maintained at 20 ± 2°C and humidity was kept at 34 ± 2%.

### Early life stress

**Figure 1:**
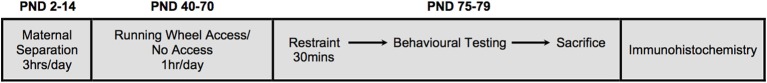
**A schematic illustration of the experimental design.** Neonatal treatment consisted of either early life stress (maternal separation) for 3hrs/day from postnatal days (PND) 2-14, or no early life stress. A subgroup of animals was given access to running wheels for 1 hr/day, 5days/week from PND40-70. All animals were subjected to restraint stress in adulthood (PND75-79) for 30 min. Immediately following restraint, animals underwent behavioral testing in the open field test (10 min) and elevated plus maze (5 min). Ninety minutes following restraint stress, animals were euthanized and brains collected.

An overview of the experimental design is outlined in Figure 1. The maternal separation procedure was performed as per previously published procedures in our laboratory (Nakamura et al., 2011), that were based on earlier studies (Plotsky and Meaney, 1993). Briefly, from PND2-14, litters in the ELS condition were removed from their home cage and individually placed in clear separation containers (13 × 13 × 7 cm) in an alternate temperature controlled room (30–34°C) for 3 h each day, from 0900 to 1200 h. Pups in the control condition remained undisturbed during this period except for weekly weighing. Bedding was left undisturbed for one week after birth, after which it was changed on a weekly basis.

### Exercise

A subgroup of animals exposed to ELS (males *n* = 6; females *n* = 9) was allowed access to a running wheel located in a separate room between PND40–70 (85 × 7.5 cm, 94 × 12 cm; Transoniq; for 1hr/day, 5days/week between 1800 and 2100 h). Only animals exposed to ELS were given access to exercise wheels, as pilot studies indicated that wheel running had no behavioral consequences for animals not exposed to ELS. (i.e., ELS+exercise group did not differ significantly from no-ELS+exercise; see Supplementary Material 1). A rotation counter attached to each wheel quantified distance traveled. Food intake was estimated across all groups during the exercise period by weighing food daily and dividing the change in food weight by the number of animals per cage.

### Adult stress exposure

Pilot studies revealed that maternal separation had no effect on open field (OF) behavior in the absence of an additional stressor in adulthood (see Supplementary Material 2). As such, between PND75-79, all animals were exposed to 30 min restraint stress prior to behavioral testing. Animals were removed from their home cage and were placed inside a soft wire mesh restrainer (25 × 20 cm) that was folded around the animal and secured with butterfly clips. This procedure has been previously demonstrated to produce a pattern of Fos-activity centered on amygdaloid and brainstem catecholamine nuclei that is distinct from physical stressors (Dayas et al., 1999). Females were tested only in the diestrous phase, monitored using a rat vaginal impedance device (Muromachi Kikai, Tokyo), as described elsewhere (Walker et al., 2010).

### Behavioral testing

Both OF and EPM testing was conducted in darkness using infrared lighting. Time and event data for both apparatuses was recorded using a computer-automated behavioral tracking system (Motion Mensura Ltd., Australia). Immediately following restraint stress, animals were placed in a square 1 × 1 m open field task apparatus enclosed by 40 cm high walls for 10 min. Exploratory variables measured were total distance traveled and time in immobility. Immediately following OF testing, approximately half of the animals (males *n* = 18; females *n* = 24) were tested on the EPM apparatus whilst the remaining animals (males *n* = 16; females *n* = 15) were returned to their home cage. Animals were transferred to a separate room where they were placed on an EPM apparatus. The EPM was painted black, and consisted of two open and two closed arms (45 cm length × 10 cm width) as well as a central square (10 × 10 cm). The primary measures on this assay included the time spent in the open arms, an index of anxiety-related behavior, and the number of closed arm entries, a measure of overall locomotor activity (Richardson et al., 2006). We also measured number of entries into the open arms and center square, as well as latency to enter the open and closed arms. Importantly, EPM-challenged animals did not differ from non-EPM-challenged animals in terms of Fos-protein expression in any of the regions studied, and therefore data from these animals were combined.

### Brain tissue harvesting and immunohistochemistry

Two hours following the initiation of restraint stress (1hr 20min following OF; 1hr 15min following EPM), rats were deeply anesthetized with sodium pentobarbitone (200 mg/kg; i.p.; Virbac, Australia). Animals were then perfused with 200 mL of 0.1 M Phosphate Buffered Saline followed by 500 mL of 4% paraformaldehyde (pH 9.5). Brains were removed and postfixed in 4% paraformaldehyde at 4°C overnight and then stored in 12.5% sucrose until sectioning. Serial rostral forebrain (40-μm) and caudal midbrain (50-μm) sections were cut using a freezing microtome (Leica Microsystems, SM2000R) and a 1-in-4 series of all sections were processed for immunohistochemical detection of Fos-protein (72 h, 1:5000, rabbit polyclonal, Santa Cruz Biotechnology, CA, USA) as described previously in detail (Smith and Day, 1993; Dayas et al., 2008). Hypothalamic sections were dual-labeled for orexin A (48 h, 1:15000, Orexin A antibody, goat polyclonal, Santa Cruz Biotechnology) or in the case of ventral tegmental area (VTA) sections, tyrosine hydroxylase (TH; 48 h, 1:10000, TH antibody, mouse polyclonal, Millipore). An equal number of animals from each treatment group were included in each individual immunohistochemistry run.

Bilateral counts of single-labeled Fos-positive cells were made in the perifornical area (PFA) and lateral hypothalamus (LH; bregma −2.28 to −3.24), paraventricular thalamus (PVT; −2.76 to −3.24) and medial parvocellular PVN (mpPVN; −1.46 to −1.94). Fos-only cell counts in the PVN and PVT were quantified using Metamorph Imaging System Software (Version 7.5; Molecular Devices Analytical Technologies) at 10× total magnification (Olympus CX40). The number of Fos-positive cells was determined by creating a region of interest around each structure and a thresholding procedure was used to quantify Fos expression. Counts of Fos-positive orexin neurons in the LH and Fos-positive TH cells in the VTA (−5.30 to −5.94) were made by one observer blind to treatment using a 20× objective (Olympus CX40). In the LH, cell counts were made in the PFA and the LH divisions, as these sections have previously been shown to contain the highest concentration of orexin neurons (Dayas et al., 2008). The PFA was defined as the area surrounding the fornix and the LH was defined as the area from the lateral side of the PFA to the optic tract (Laorden et al., 2012). Cells in the VTA were quantified in the parabrachial pigmented nucleus (PBP) region of the VTA. All brain coordinates were based on the Paxinos and Watson atlas (Paxinos and Watson, 2007).

### Data analysis

All statistical analyses were conducted using IBM SPSS V19. Male and female animals were analyzed separately. ANCOVA revealed no significant effect of litter size and male to female ratio for all comparisons. Body weight of treatment groups was compared on PND72 using a one-way between-subjects ANOVA. Food intake and behavioral data were compared across treatment groups using a one-way between-subjects ANOVA and subsequent least significant differences (LSD) *post-hoc* analyses where appropriate. For immunohistochemical analyses, all cell counts were averaged across each animal for each rostrocaudal level of each brain region examined. To minimize the effects of variability across multiple immunohistochemistry runs, counts for each treatment group were calculated as a fold change relative to control animals processed in the same run. These fold changes were averaged across the rostral-caudal extent of each brain region and were compared across groups using one-way ANOVAs. These analyses were followed by LSD *post-hoc* analyses where appropriate. An alpha value of 0.05 was adopted for all statistical tests. All figures depict means and standard errors.

## Results

### Effect of ELS on body weight and food intake

**Figure 2:**
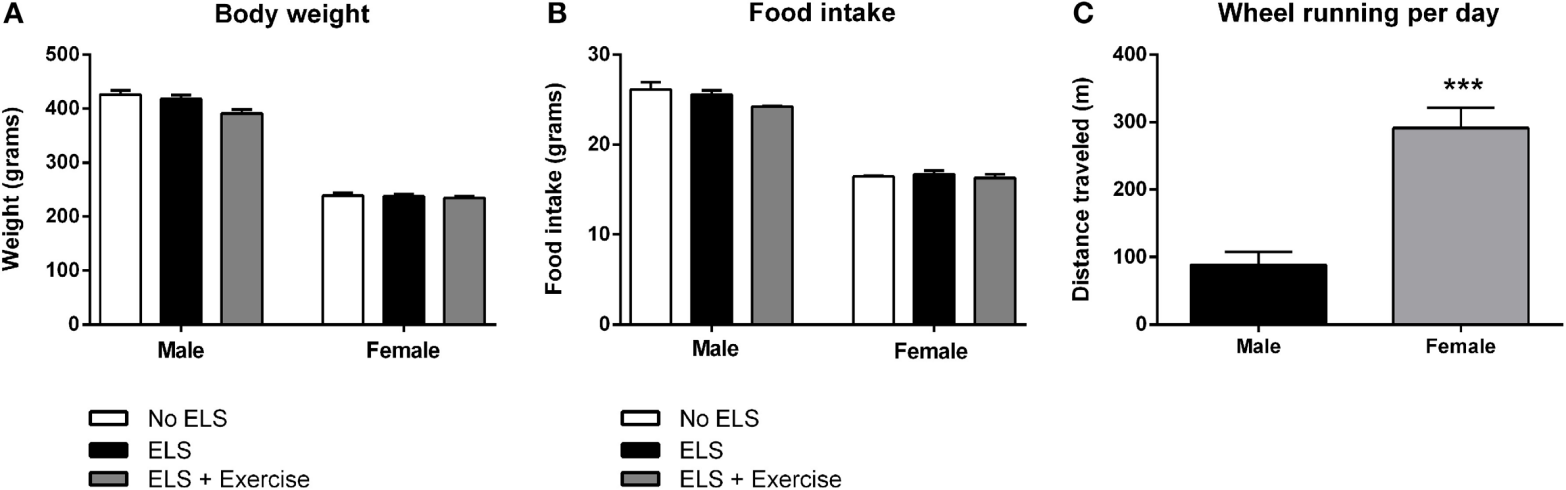
**Effect of early life stress (ELS) on body weight and food intake; and sex differences in wheel running.** There was no effect of treatment on body weight at postnatal day 72 in both male and female rats. Males: No ELS: *n* = 13; ELS: *n* = 15; ELS + Ex: *n* = 6. Females: No ELS: *n* = 16; ELS: *n* = 14; ELS + Ex: *n* = 9 **(A)**. Similarly, ELS had no effect on food intake. Males: No ELS: *n* = 6; ELS: *n* = 6; ELS + Ex: *n* = 5. Females: No ELS: *n* = 7; ELS: *n* = 7; ELS + Ex: *n* = 9 **(B)**. Female rats engaged in significantly greater amounts of wheel running per day compared to male rats. Males: *n* = 6; Females: *n* = 9 **(C)**. ^***^p < 0.001.

On PND72, male animals from each treatment group did not differ significantly in terms of their body weight [*F*_(2, 31)_ = 2.366, *p* = 0.106], or food intake across the experimental period [*F*_(2, 14)_ = 2.554, *p* = 0.113]. Similarly, body weight of females was indistinguishable between treatment groups [*F*_(2, 36)_ = 0.026, *p* = 0.975] as was their food intake [*F*_(2, 20)_ = 0.302, *p* = 0.743]. Interestingly, wheel rotations were on average approximately three times higher in females than male animals in each exercise session [*F*_(1, 13)_ = 19.429, *p* < 0.001; Figure 2].

### ELS was associated with a reduced percentage of Fos-positive orexin cells after psychological stress: protective effect of exercise only in males

**Figure 3:**
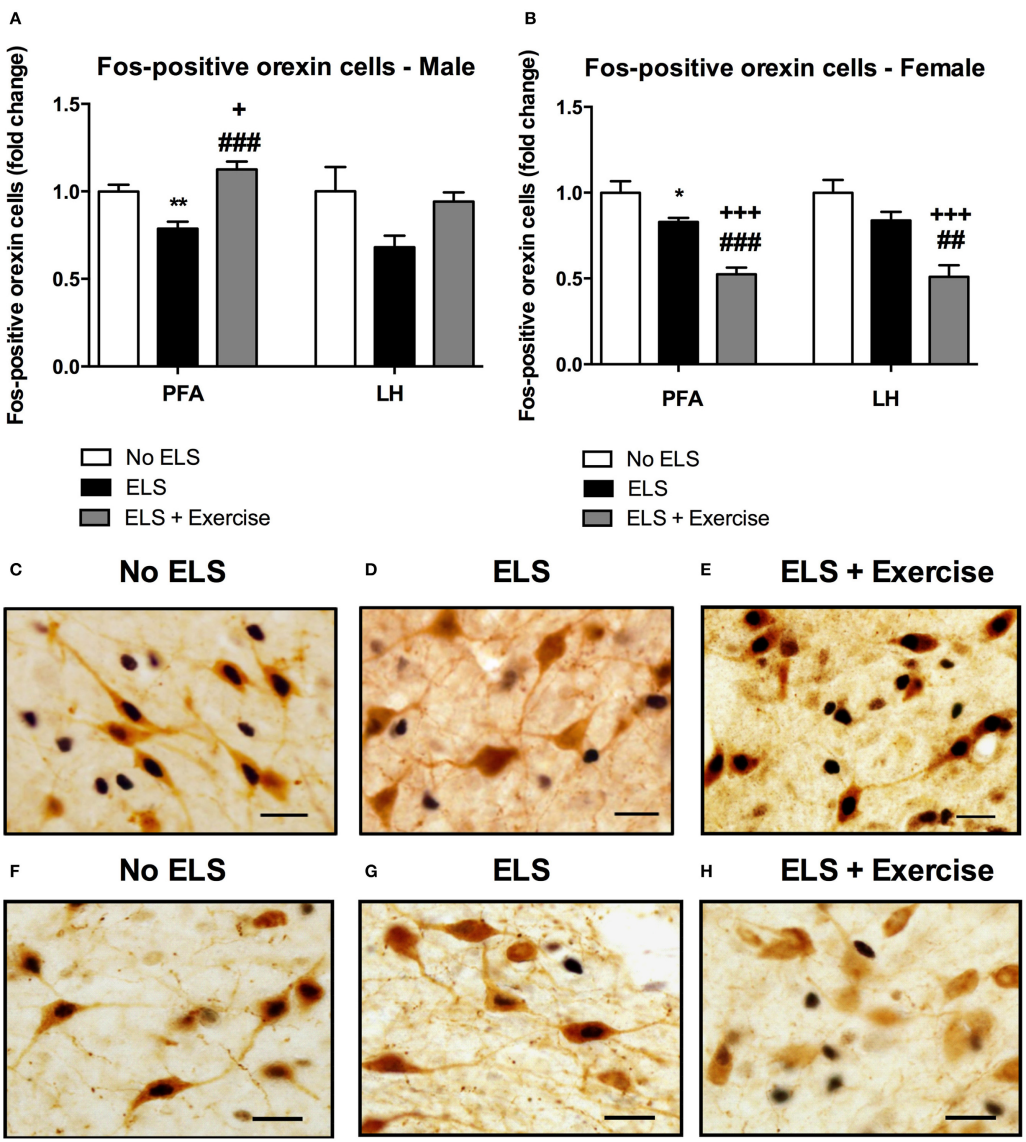
**Early life stress (ELS) was associated with a decrease in the percentage of Fos-positive orexin cells in both male and female rats: Wheel running protected against these effects in males, but exaggerated these effects in females.** The percentage of Fos-positive orexin cells in the PFA of the hypothalamus was significantly lower in male ELS rats compared to controls. Wheel running was protective against the effects of ELS in this region. This trend was also observed in the LH but failed to reach significance (*p* = 0.06; **A**). As in male animals, ELS-exposed females exhibited a reduced percentage of Fos-positive orexin cells in the PFA. In contrast to males, wheel running exacerbated these effects in female rats **(B)**. A similar trend was observed in the LH **(B)**. Photomicrographs of coronal sections of the PFA of the hypothalamus immunolabeled for Fos-protein and orexin in males **(C,D,E)** and females **(F,G,H)**. Males: No ELS: *n* = 7; ELS: *n* = 9; ELS + Ex: *n* = 6. Females: No ELS: *n* = 7; ELS: *n* = 8, ELS + Ex: *n* = 7. ^*^p < 0.05 vs. No ELS, ^**^p < 0.01 vs. No ELS, ^+^*p* < 0.05 vs. No ELS, ^+++^*p* < 0.001 vs. No ELS, ^##^*p* < 0.01 vs. ELS, ^###^*p* < 0.001 vs. ELS, scale bar, 20 μm.

In male rats there was no effect of treatment on the number of orexin immunoreactive cells in either the PFA or LH subdivisions of the hypothalamus [*F*_(2, 18)_ = 0.292, *p* = 0.750; *F*_(2, 18)_ = 1.648, *p* = 0.220 respectively, data not shown]. To assess the effect of ELS on the reactivity of orexin neurons to stress in adulthood, we quantified the percentage of orexin cells expressing Fos-protein following psychological stress. ANOVA revealed a significant effect of treatment on the percentage of orexin cells expressing Fos protein in the PFA [*F*_(2, 18)_ = 17.646, *p* < 0.001], and a trend toward significance in the LH [*F*_(2, 18)_ = 3.248, *p* = 0.062]. *Post-hoc* analyses revealed that ELS animals displayed a significantly lower percentage of orexin neurons that expressed Fos-protein after psychological stress compared to controls in the PFA (*p* = 0.002). Interestingly, ELS animals given access to running wheels displayed a pattern of Fos/orexin immunoreactivity in the PFA that was significantly greater than that of other treatment groups (*p* = 0.042 compared to controls, *p* < 0.001 compared to ELS; Figure 3).

Similar to males, orexin cell numbers did not differ across treatment groups in female rats in both the PFA and LH [*F*_(2, 18)_ = 0.141, *p* = 0.87; *F*_(2, 18)_ = 0.166, *p* = 0.849, respectively; data not shown]. There was a significant main effect of treatment on the percentage of orexin cells that displayed Fos-like immunoreactivity in response to restraint stress in the PFA [*F*_(2, 18)_ = 26.907, *p* < 0.001] and LH [*F*_(2, 18)_ = 14.292, *p* < 0.001]. Consistent with male animals, *post-hoc* analyses showed that ELS females exhibited a significantly lower percentage of Fos-positive orexin cells compared to control animals in the PFA (*p* = 0.018) and a similar trend in the LH (*p* = 0.094). In contrast to males however, access to running wheels tended to exacerbate the effect of treatment on orexin cell reactivity as assessed by Fos-labeling in the PFA (*p* < 0.001 compared to controls and ELS) and LH (*p* < 0.001 compared to controls, *p* < 0.01 compared to ELS; Figure 3).

### ELS was associated with a reduction in Fos-protein expression in PVN and PVT neurons following psychological stress: protective effect of exercise in male but not female rats

**Figure 4:**
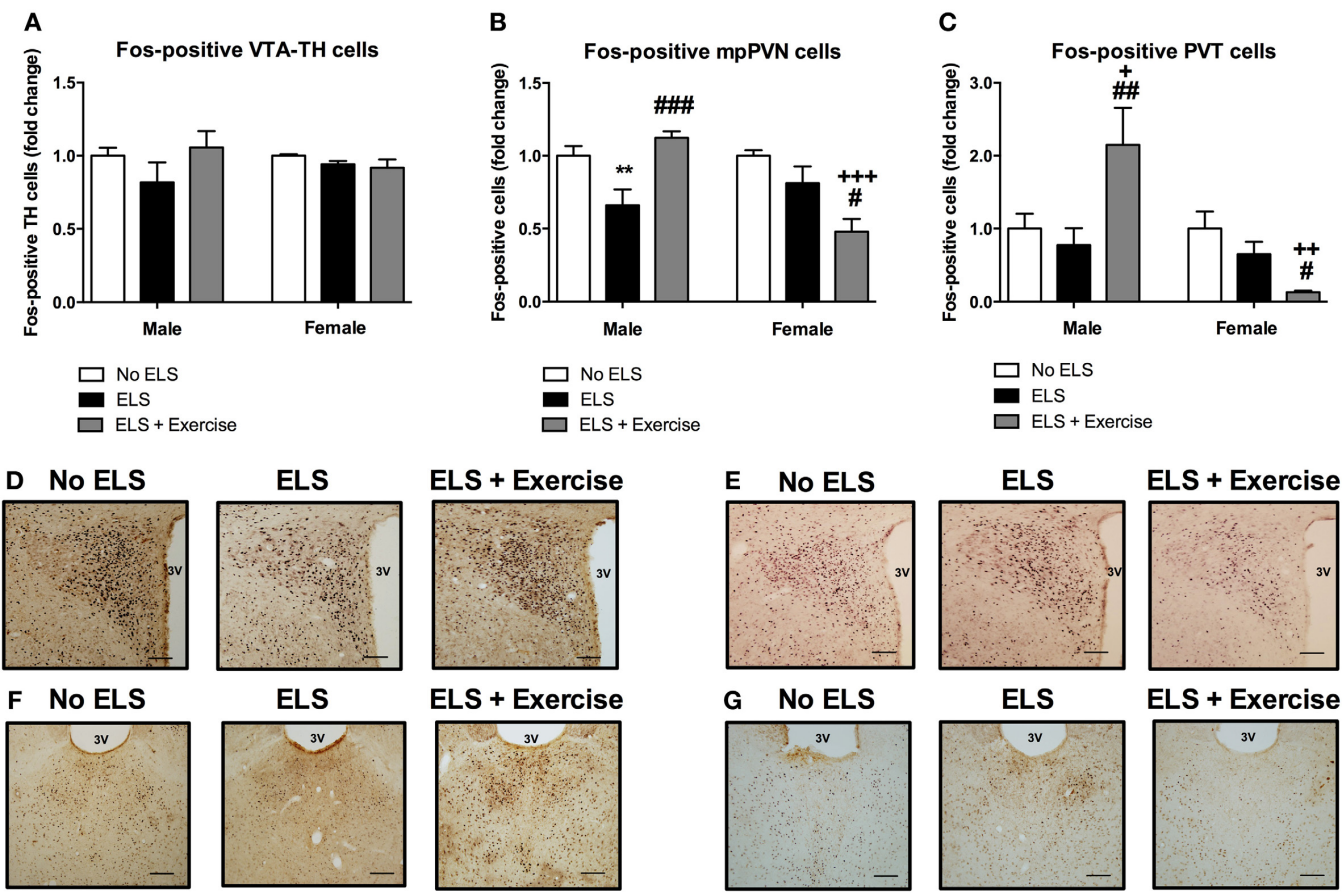
**Early life stress (ELS) was associated with a decrease in Fos-immunoreactivity in mpPVN and PVT neurons: Protective effect of exercise in male but not female rats.** ELS resulted in a non-significant reduction in the percentage of Fos-positive TH cells in the VTA in male rats. Wheel running appeared to be protective against these effects. No ELS: *n* = 6; ELS: *n* = 6, ELS + Ex: *n* = 6 **(A)**. In females, there was no effect of ELS or exercise on the percentage of Fos-positive TH cells in the VTA. No ELS: *n* = 7; ELS: *n* = 7; ELS + Ex: *n* = 7 **(A)**. In the PVN, there was an ELS-induced reduction in Fos-positive cells in males and this effect was reversed by wheel running. There was no effect of ELS on the number of Fos-positive cells in the PVT in males however, wheel running was protective against the effects of ELS. No ELS: *n* = 7; ELS *n* = 9; ELS + Ex: *n* = 6 **(B,C)**. In females, there was no effect of ELS on the number of Fos-positive cells in the PVN and PVT however, wheel running did exacerbate the effects of ELS. No ELS: *n* = 7; ELS: *n* = 8; ELS + Ex: *n* = 7 **(B,C)**. Coronal sections of the PVN (males: **D**; females: **E**) and PVT (males: **F**; females: **G**) immunolabeled for Fos-protein, scale bar 100 μm. ^**^p < 0.01 vs. No ELS, ^+^*p* < 0.05 vs. No ELS, ^++^*p* < 0.01 vs. No ELS, ^+++^*p* < 0.001 vs. No ELS, ^#^*p* < 0.05 vs. ELS, ^##^*p* < 0.01 vs. ELS, ^###^*p* < 0.001 vs. ELS.

In addition to orexin neurons we assessed the level of Fos-like immunoreactivity in the VTA, PVN and PVT following restraint stress in adulthood. In males, the percentage of Fos-positive TH cells in the VTA did not differ significantly between treatment groups [*F*_(2, 15)_ = 1.369, *p* = 0.284; Figure 4]. There was a significant main effect of treatment on Fos-immunoreactivty in the PVN [*F*_(2, 15)_ = 9.316, *p* = 0.002], with *post-hoc* analyses revealing a significant reduction in Fos-positive cells in ELS animals compared to controls (*p* = 0.008). Access to voluntary exercise significantly increased the number of Fos-positive PVN cells compared to ELS-exposed animals (*p* < 0.001). There was no significant difference between exercised males and controls in this region (*p* = 0.287; Figure 4). In the PVT, there was a significant main effect of treatment on Fos-positive cells [*F*_(2, 19)_ = 5.248, *p* = 0.015]. *Post-hoc* analyses revealed a significant increase in Fos-immunoreactivity in the PVT of animals given access to running wheels (*p* = 0.023 compared to controls, and *p* = 0.006 compared to ELS; Figure 4). No significant difference was found in the number of Fos-positive cells in the ELS group compared to controls in this region (*p* = 0.602).

In females, there was no significant main effect of treatment on the number of TH-positive cells that expressed Fos-protein [*F*_(2, 18)_ = 1.415, *p* = 0.269; Figure 4]. In the PVN, ANOVA revealed a significant main effect of treatment on the number of Fos-positive cells [*F*_(2, 19)_ = 8.27, *p* = 0.003]. *Post-hoc* analyses revealed no significant difference between controls and ELS (*p* = 0.152). However, access to running wheels significantly reduced the number of Fos-positive PVN cells (*p* < 0.001 compared to controls, *p* = 0.016 compared to ELS; Figure 4). In the PVT, there was a significant main effect of treatment on Fos-protein expression [*F*_(2, 19)_ = 6.409, *p* = 0.008]. *Post-hoc* analyses revealed that there was no significant difference in the number of Fos-positive cells in ELS animals compared to controls (*p* = 0.156). A significant reduction in Fos-positive PVT cells was observed in rats given access to running wheels (*p* = 0.002 compared to controls, *p* = 0.041 compared to ELS; Figure 4).

### ELS animals had lower exploratory behavior in the open field following psychological stress in adulthood: protective effect of exercise in male but not female rats

**Figure 5:**
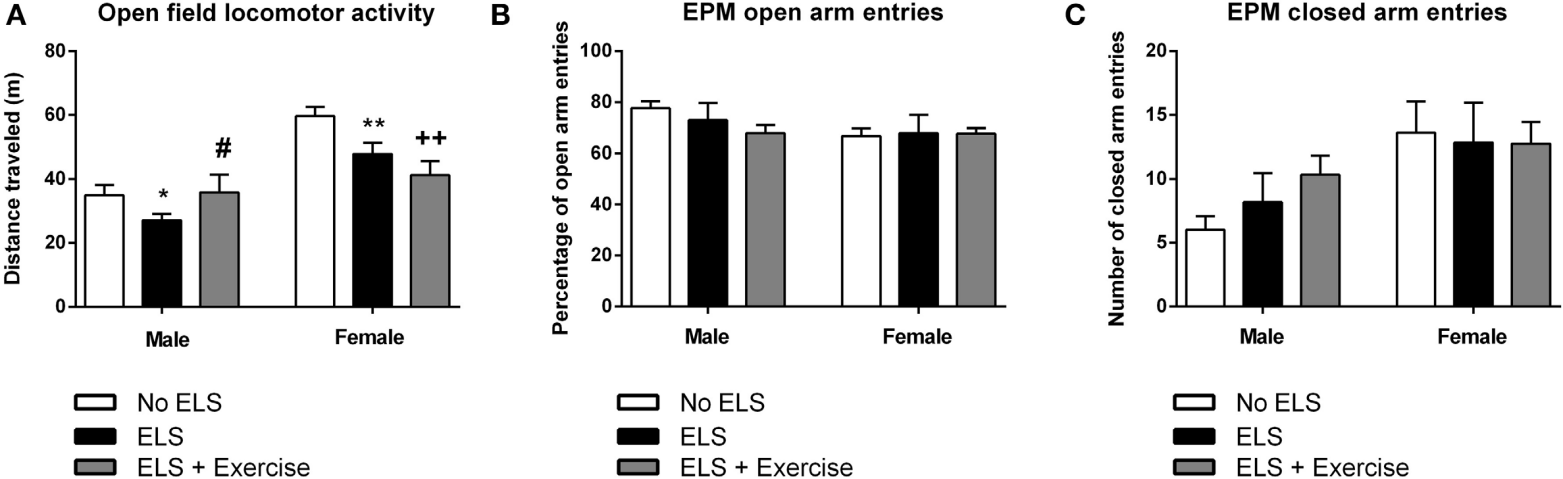
**Early life stress (ELS) is associated with reduced locomotor activity in the open field task: Protective effects of exercise in males, but not in females.** In both males and females, ELS was associated with a significant reduction in the distance traveled in the open field. Wheel running protected against this effect in male rats, but exacerbated the ELS effect in female rats **(A)**. In both male and female rats, there was no effect of treatment or wheel running on the number of open **(B)** or closed **(C)** arm entries across in the elevated plus maze. Males: No ELS: *n* = 13; ELS: *n* = 15; ELS + Ex: *n* = 6. Females: No ELS: *n* = 16; ELS: *n* = 14; ELS + Ex: *n* = 9. ^*^p < 0.05 vs. No ELS, ^**^p < 0.01 vs. No ELS, ^++^*p* < 0.01 vs. No ELS, ^#^*p* < 0.05 vs. ELS.

In males, one-way ANOVA revealed a main effect of treatment on the distance traveled in the OF [*F*_(2, 31)_ = 2.66, *p* = 0.043]. *Post-hoc* comparisons revealed that ELS-exposed animals traveled significantly less distance compared to controls (*p* = 0.026). This effect was reversed when ELS animals were given access to voluntary exercise throughout adolescence (*p* = 0.044, compared to ELS group; Figure 5). Analyses also showed a trend toward the ELS group exhibiting increased time spent in immobility as compared to the control group, with this effect again being reversed by exercise intervention [*F*_(2, 31)_ = 2.21, *p* = 0.063, data not shown).

In females, there was a significant main effect of treatment in terms of distance traveled [*F*_(2, 36)_ = 7.13, *p* = 0.001]. Similar to males, maternally separated animals exhibited locomotor hypoactivity in the OF when compared to controls (*p* = 0.007). In contrast to males, this effect was not reversed by voluntary exercise, but in fact was exaggerated (*p* < 0.001, compared to controls; Figure 5). This same trend was observed in terms of time spent in immobility [*F*_(2, 36)_ = 7.44, *p* = 0.001] with ELS females spending significantly more time in immobility compared to controls (*p* = 0.01). Time spent in immobility was exaggerated in exercised females (*p* < 0.001 compared to controls, data not shown).

With respect to the EPM, there was no effect of treatment on the number of open [*F*_(2, 15)_ = 0.382, *p* = 0.689] or closed [*F*_(2, 15)_ = 1.624, *p* = 0.230] arm entries in males. Similarly, in females there was no difference between treatment groups on open [*F*_(2, 21)_ = 0.617, *p* = 0.549] or closed arm entries [*F*_(2, 21)_ = 0.040, *p* = 0.961; Figure 5]. Additionally, there was no effect of treatment on the duration spent in the open arms, closed arms or the center square, or the latency to enter the open or closed arms in either males or females (*p*'s > 0.05).

## Discussion

In the present study we report that ELS-exposed male and female rats exhibited a “hypoactive” orexin cell response to restraint stress, as assessed by Fos-like immunoreactivity, particularly in the PFA. Notably, both male and female animals exposed to ELS displayed reduced exploratory behavior on the OF following restraint stress. Interestingly, the ability of exercise to ameliorate ELS-induced deficits was strongly sex-dependent. A similar level of sex-specificity was also seen in brain regions that are known to respond to orexin innervation. Together these results not only highlight the profound effect that ELS has on orexin function in adulthood but also the positive effects of exercise on this deficit and how this differs across sexes.

The primary aim of this study was to assess the impact of ELS on subsequent orexin cell reactivity to psychological stress in adulthood. Due to known sex differences in neural responses to stress, we examined the degree to which orexin cells had become activated by quantifying the number of orexin cells that were Fos-positive following psychological stress in adulthood in both male and female rats. Using this well-characterized strategy, orexin function in both males and females that were exposed to ELS was substantially lower than non-ELS controls. ELS-exposed animals also exhibited significantly lower activity in the OF. These findings are interesting in light of recent findings from other preclinical studies that have shown that chronic stress results in reduced orexin system function and increased depressive-like behavior (Lutter et al., 2008; Nocjar et al., 2012). Further, recent human studies have reported an inverse relationship between CSF orexin peptide levels and symptoms of depression (Brundin et al., 2007, 2009). With these findings in mind, it is possible that in our study, reduced orexin activity induced by ELS resulted in a depressive-like behavioral state that manifested as reduced exploratory behavior. Future studies should assess whether these changes in orexin cell function also manifest as deficits in motivated behavior on behavioral assays such as the sucrose preference test and/or forced swim test. Further investigation is also warranted to understand the relevance of our observation that orexin hypoactivity was more pronounced in the PFA compared to the LH, as separate functions have been ascribed to these populations (stress reactivity and reward-seeking, respectively; Harris and Aston-Jones, 2006).

Perhaps the most striking observation of the present study was that behavioral deficits associated with ELS were not observed in male rats allowed access to voluntary exercise. These findings are consistent with previous studies demonstrating that voluntary wheel running protects against the expression of anxiety-like behavior in adult male rats exposed to maternal separation stress (Maniam and Morris, 2010) or footshock stress in adulthood (Greenwood et al., 2013). Given that there was a significant “wash-out” period between wheel running and restraint, it is likely that exercise reversed ELS-induced changes in LH-orexin circuit function rather than prevented the acute effects of restraint. However, we acknowledge further tests are required to address this issue. With respect to the sex-specific effects we observed, our findings are in line with those of Brocardo et al. (2012) who showed that voluntary exercise had no effect on the expression of anxiety- and depression-like behaviors in female rats exposed to ethanol in early life, despite this intervention having protective effects in males (Brocardo et al., 2012). Similarly, findings from the addiction field have yielded differential effects of voluntary exercise on drug-related behaviors in males and females, despite being exposed to identical exercise regimes (Smith et al., 2008; Ehringer et al., 2009; Thanos et al., 2010). Our findings that exercise actually tended to exacerbate ELS-induced orexin and behavioral changes in females perhaps points to the possibility that the increased wheel running observed in females was actually stress provoking. Exercise-induced corticosterone secretion may have subsequently influenced orexin cell responsivity (Ford et al., 2005). These findings point to the need for a greater understanding of how exercise conditions (type, intensity, duration) can be modified to produce beneficial effects in both sexes.

Interestingly, we observed a similar pattern of reactivity in key orexinergic targets, including the mpPVN, PVT, and VTA dopamine neurons. With respect to the mpPVN, there is now considerable evidence that orexin directly modulates the neuroendocrine response to stress. The mpPVN is densely innervated by orexinergic terminals and mainly expresses orexin receptor 2 (Peyron et al., 1998; Trivedi et al., 1998). Further, central administration of orexin-A induces Fos-protein expression in corticotropin releasing factor (CRF)-expressing cells in the mpPVN (Sakamoto et al., 2004) and increases plasma corticosterone and adrenocorticoptropin hormone (ACTH) levels (Ida et al., 2000; Kuru et al., 2000). Whilst we did not directly measure HPA-axis activity, maternal separation stress has previously been shown to be associated with impaired mpPVN and HPA-axis responsivity to stress in adulthood (Plotsky and Meaney, 1993; Ladd et al., 2000; Daniels et al., 2004). Further studies are required to assess whether this impairment is directly associated with the reduction in orexin function observed here. With respect to the PVT, this region is also known to be densely innervated by orexin terminals (Kirouac et al., 2005) and contains high densities of both orexin receptors (Marcus et al., 2001). Orexin signaling in the PVT has recently been shown to be important for both the neuroendocrine response to stress (Heydendael et al., 2012) and the expression of stress-related behaviors (Li et al., 2010a,b; James and Dayas, 2013; Yeoh et al., 2014). Further, stimulation of the PVT can modulate dopamine release in the nucleus accumbens (NAC; Jones et al., 1989; Parsons et al., 2007). Reduced PVT signaling in ELS animals may therefore contribute to reduced striatal dopamine release, an outcome consistent with previous studies showing that hypoactivity of the VTA dopamine-NAC projection is causally linked to depressive-like behavior following social defeat (Berton et al., 2006).

One caveat of our experimental design is that OF and EPM testing were performed after restraint stress. While we attribute the pattern of Fos-protein expression observed as a response to restraint stress, given the time course typically required for maximal Fos-protein induction (2 h; Kovács, 1998), we cannot exclude the possibility that OF and EPM testing also influenced the expression of this immediate early gene. Regardless, these challenges are typically regarded as psychological stressors, which produce similar patterns of Fos-protein expression in stress-sensitive brain regions as restraint stress (Dayas et al., 2001). Further, no differences were observed in terms of Fos-protein expression amongst EPM-challenged vs. non-EPM-challenged animals, suggesting that behavioral testing did not have any confounding effects on Fos expression. It is also important to note that a previous report failed to observe an increase in Fos-protein expression in orexin-positive neurons in response to restraint (Furlong et al., 2009). These experiments however, were carried out in animals with no prior stress exposure, and the effects observed in our study may reflect a more important role for orexin signaling in stress reactivity in chronically stressed animals.

In summary, the present study provides novel evidence that the orexin system's response to adult stress is altered by ELS. Identical effects of ELS on orexin cell activity in stressed adults were observed in the PFA of male and female rats. These data are consistent with recent clinical evidence indicating that vulnerability to stress-related mood disorders is linked with orexin system hypofunction (Brundin et al., 2007, 2009). We also show that exercise was protective against both the behavioral (OF activity) and neural effects of ELS in male rats, suggesting that the beneficial effects of exercise on stress-related behavior is associated with a “normalization” of orexin function and that, under some conditions, the orexin system can be modified by non-pharmacological methods. Surprisingly, female rats exhibited significantly greater deficits in orexin function following wheel running, suggesting that while the effects of ELS on orexin function are similar across sexes, future studies will need to consider alternative approaches to recover orexin function in female rats. These findings extend recent studies showing that the orexin system is highly plastic and is readily modified by environmental events (Yeoh et al., 2012). More broadly, this study highlights the importance of studying sex-based differences in stress-related pathology (Clayton and Collins, 2014).

### Conflict of interest statement

The authors declare that the research was conducted in the absence of any commercial or financial relationships that could be construed as a potential conflict of interest.
