# Supplementary material for: Exercise reverses the effects of early life stress on orexin cell reactivity in male but not female rats
Source: Front Behav Neurosci. 2014 Jul 23;8:244. doi: 10.3389/fnbeh.2014.00244 (PMC4107856; doi:10.3389/fnbeh.2014.00244)
Supplement: Supplementary file 1 [file Presentation1.PDF]

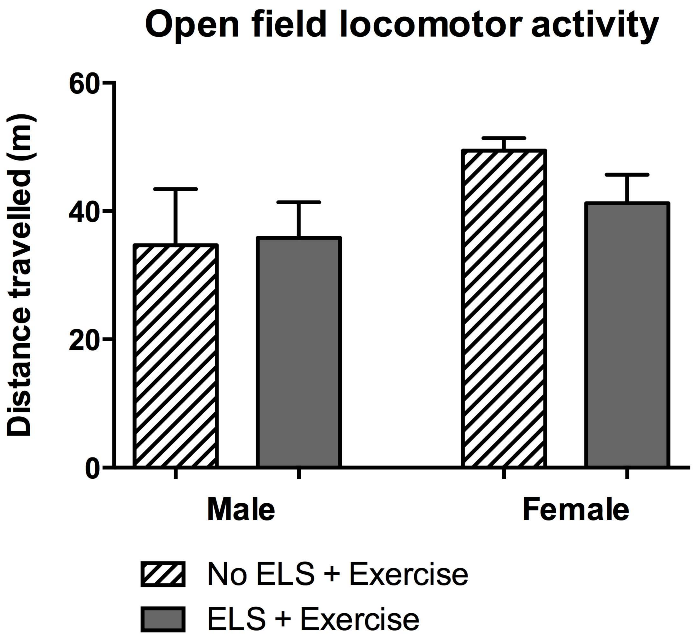

Figure S1. Wheel running had no behavioral consequences for rats not exposed to ELS. In both male and female rats, access to running wheels did not result in a significant change in locomotor activity in the open field test in animals not exposed to ELS, as compared to those that were exposed to ELS.

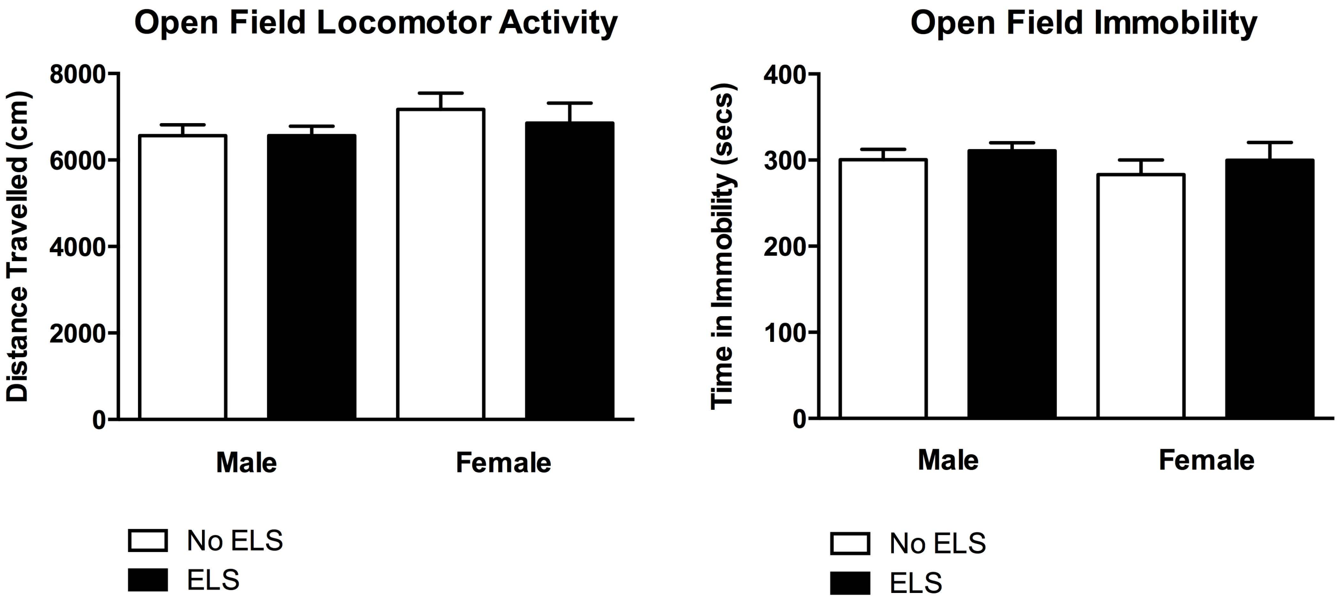

Figure S2. There was no effect of early life stress (ELS) on open field locomotor activity in the absence of an additional stressor in adulthood. In both male and female rats, exposure to maternal separation stress had no effect on locomotor activity (exploration) or time spent immobile in the open field test when animals were not exposed to an additional psychological stressor (restraint) in adulthood. Based on these findings, all animals were exposed to restraint stress prior to behavioural testing in adulthood.
